# Roles of EphA2 in Development and Disease

**DOI:** 10.3390/genes4030334

**Published:** 2013-07-01

**Authors:** Jeong Eun Park, Alexander I. Son, Renping Zhou

**Affiliations:** Susan Lehman-Cullman Laboratory for Cancer Research, Department of Chemical Biology, Ernest Mario School of Pharmacy, Rutgers University, Piscataway, NJ 08854, USA; E-Mails: jeparkk@rci.rutgers.edu (J.E.P.); szero013@gmail.com (A.I.S.)

**Keywords:** EphA2, ephrin, development, cataract, kidney, bone, ear, mammary gland

## Abstract

The Eph family of receptor tyrosine kinases (RTKs) has been implicated in the regulation of many aspects of mammalian development. Recent analyses have revealed that the EphA2 receptor is a key modulator for a wide variety of cellular functions. This review focuses on the roles of EphA2 in both development and disease.

## 1. Introduction

Receptor tyrosine kinases (RTKs) are membrane spanning proteins that play an important role in a wide range of signaling processes in development [1,2]. The largest group of RTKs is the Eph family of molecules, which comprises of 14 members (EphA1–EphA8, EphA10; EphB1–EphB4, EphB6) in mammals [3,4,5,6]. Eph receptors and their ligands, the ephrins, have been shown to play several key roles in embryonic development [7,8], including tissue boundary formation [9,10,11], neural crest cell migration [12,13,14], axon guidance [15,16], central nervous system patterning [17,18], bone remodeling [19,20,21,22] and vascular organization [23,24,25,26,27,28,29]. In general, Eph and ephrin proteins are highly expressed during development while declining in later stages [30]. However, there is increasing evidence that these molecules can be re-expressed in specific circumstances such as tumorigenesis [30,31].

Recent investigations have shown that EphA2 has many important and diverse biological functions. EphA2 expression has been detected in a wide assortment of tissues including the brain, skin, bone marrow, lung, thymus, spleen, liver, small intestine, colon, urinary bladder, kidney, uterus, testis and prostate [32]. Most tissues express low levels of EphA2. Overexpression and dysregulation of this receptor have been associated with carcinogenesis, metastasis, and poor clinical prognosis [31,33,34]. In addition, EphA2 has attracted special attention in the field of lens [35,36,37,38,39,40,41], kidney [42,43], bone [22], mammary gland [34,44,45,46,47], and ear [48] development. Here, we highlight recent findings addressing the crucial roles of EphA2 receptor and ephrin-A ligands in tissue morphogenesis and disease. This review focuses on EphA2 functions that have not been reviewed previously, and its roles in tumorigenesis and cancer are not discussed since the several acceptant reviews in this area have been published recently [31,49,50,51,52,53,54,55,56].

## 2. Eph Receptors and Ephrins

The Eph receptors are named after erythropoietin-producing hepatocellular carcinoma cell lines in which the first member of the family, EphA1, was isolated [57]. Their respective ligands, the membrane-bound ephrins [58], were first identified in 1994 with the discovery of ephrin-A1 [59]. Both the Eph receptors and ephrin ligands are divided into the A (EphA and ephrin-A) and B (EphB and ephrin-B) subgroups. The categorization was based on sequence homology within subfamilies and binding affinities between Eph-ephrin pairs. In the case of the ligands, the mode of membrane attachment is different, as ephrin-A ligands are attached to the cell surface through a glycophosphotidylinositol (GPI) anchor, while the ephrin-B ligands are anchored by a hydrophobic transmembrane region [6,60].

In general, the EphA members (EphA1–EphA10) primarily interact with the ephrin-A ligands (ephrin-A1-ephrin-A6), while the EphB receptors (EphB1-EphB6) bind to the ephrin-B ligands (ephrin-B1-ephrin-B3) [5,6,60,61]. However, interactions between receptor and ligand subgroups have been previously reported; for instance, EphA4 has been shown to interact with both ephrin-B2 [62] and ephrin-B3 [63], and EphB2 has been found to be activated by ephrin-A5 [64].

### 2.1. Domain Configuration

**Figure 1:**
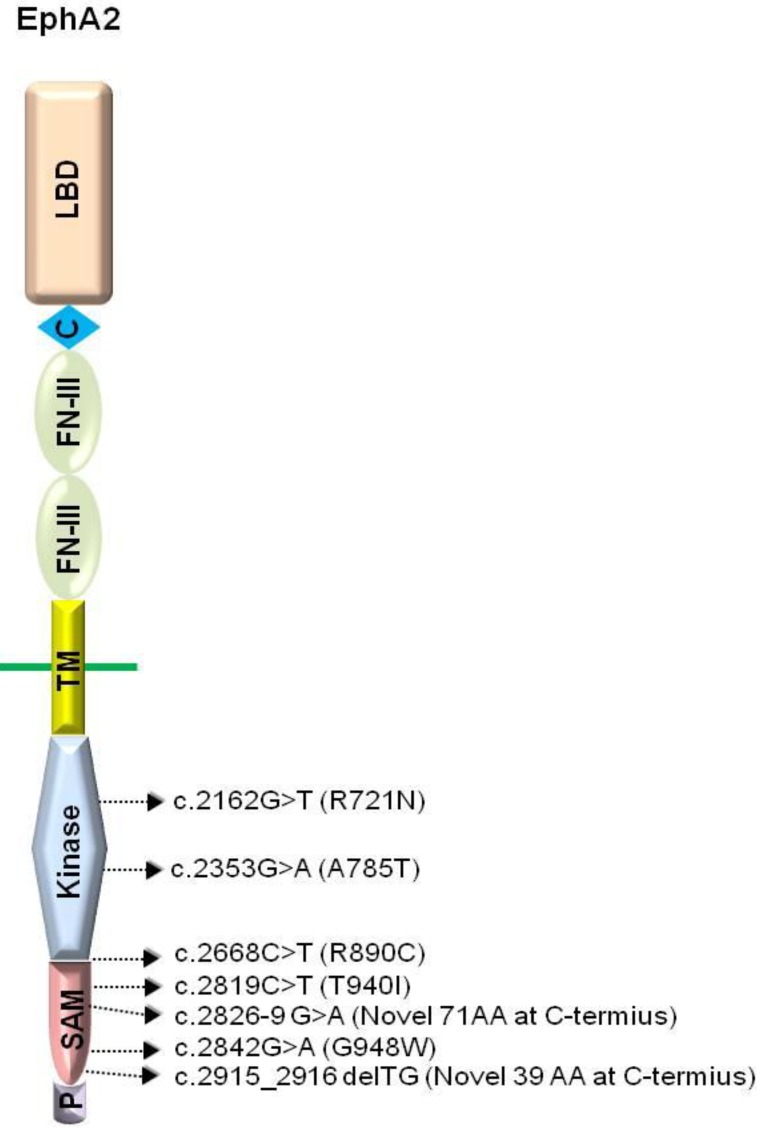
Domain structure of EphA2 and the locations of human cataract mutations. LBD, ligand binding domain; C, cystein rich; FN-III, fibronectin type-III domain; TM, transmembrane domain; Kinase, protein tyrosine kinase domain; SAM, sterile alpha motif domain; P, PDZ-binding motif. Arrows indicate relative location of human cataract mutations.

The Eph receptors comprise several distinctive domains required for their signaling capabilities. The extracellular domain contains an ephrin ligand-binding domain in its *N*-terminal-most region, followed by a cysteine-rich region and two fibronectin type-III repeats [65]. The intracellular region comprises of the signaling components which include a tyrosine kinase domain, a SAM (Sterile Alpha Motif) domain, and a PDZ (Postsynaptic density protein, Disks large, Zona occludens)-binding motif (Figure 1) [65]. Both the SAM and the PDZ domains have been shown to mediate protein-protein interactions [66,67,68,69,70,71].

### 2.2. Signaling

Eph receptors regulate a diverse range of biological processes including cell proliferation, differentiation, migration and tissue morphogenesis [7,30,31,72,73,74]. Signal transduction by the Eph family is a multistep process leading to the assembly of higher-order signaling clusters in the interacting cells [75]. The Eph family is capable of bidirectional signaling; upon interaction between receptor-ligand pairs, signaling events may be initiated by receptor-expressing cells (forward signaling), ligand-expressing cells (reverse signaling), or both (bidirectional signaling) [31,74,76,77,78]. These events result in the reorganization of the actin cytoskeleton, which leads to contact-dependent cell-cell attraction or repulsion and early embryonic cell motility and migration [15,72,79,80,81,82].

A major consequence of Eph-ephrin interaction is intercellular repulsion. This function occurs, in part, by proteolytic cleavage of the ephrin ligand at the juxtamembrane domain by the ADAM-10/Kuzbanian metalloproteinase leading to the disengagement of cells [83]. Eph-ephrin signaling can also elicit endocytosis and degradation through the Cbl ubiquitin ligase [84,85,86], which binds to activated receptors and acts also as an adaptor for signal tranducers [87].

Numerous downstream molecules mediate signal transmission by activated Eph receptors, including the Src kinase family [88,89] and the Ras and Rho family of small GTP-binding proteins (G-proteins) [76,90,91,92,93,94,95,96]. EphB4 activation results in Rac activation and assembly of actin and lammelipodia formation in the cells expressing the receptor [91]. In addition, Eph receptors have been shown to interact with receptors of other families including ErbB2 [31,97]. For reverse signaling, ephrin-B ligands are capable of activating Src family kinases (SFKs) [88] and recruiting cytoplasmic SH2 and PDZ domain containing proteins, such as Grb4 adaptor protein [98], glutamate receptor interacting protein (GRIP) [99], phospho-tyrosine phosphatase PTP-BL [88] and PDZ-regulator of heterotrimeric G protein signaling (RGS) 3 [92]. Ephrin-As collaborate with the ADAM10/Kuzbanian [83], the p75 (NTR) protein [79] and the NF-κB and Ret tyrosine kinase receptors to send signals [93]. The ephrin ligands can also initiate reverse signaling through SH2 or PDZ domain-independent associations [92,98,99].

The juxtamembrane region of Eph receptors has two highly conserved tyrosine residues (Tyr^588^ and Tyr^594^ in EphA2; Tyr^596^ and Tyr^602^ in EphA4; Tyr^594^ and Tyr^600^ in EphB1; Tyr^604^ and Tyr^610^ in EphB2). These tyrosine residues are major sites for autophosphorylation [100,101,102] and interact with a number of SH2 domain-containing cytoplasmic proteins including the human growth factor receptor bound protein (Grb7) [103], the Ras GTPase-activating protein (RasGAP) [104], the p85 subunit of phosphatidylinositol 3-kinase (PI3K), the adaptor protein NCK [102,104,105], and the R-Ras- and Rap1A-interacting protein SHEP-1 [90]. These SH2 domain-containing adaptor partners can modify several aspects of cellular behavior including cell adhesion and cytoskeletal structures [90,102,103,104]. The SH2 domain of Grb7 binds to the tyrosine phosphorylated SAM domain of EphB1, and this interaction affects regulation of cell migration [103,106]. In PC-3 prostate carcinoma cells, activated EphA2 caused dissociation of focal adhesion kinase (FAK) and the transient recruitment of the phosphotyrosine phosphatase Shp2 [82]. This event also correlates with inhibition of integrin-mediated adhesion, cell spreading and cell migration. In contrast, ligand activation of EphA2 can increase FAK phosphorylation in NIH3T3 cells and enhance cell spreading in a FAK-dependent manner [82]. The mechanisms of Eph receptor forward signaling and ephrin ligand reverse signaling are complex and have been well reviewed previously [31,52,55,72,76,78,81,107,108].

## 3. EphA2

EphA2 was identified in 1990 by the screening of the human epithelial HeLa cell cDNA library using degenerate probes designed to hybridize highly conserved regions of protein tyrosine kinases [109]. Originally named epithelial cell kinase (Eck) for its expression in the majority of epithelial cells, it resides on human chromosome 1p36.1 [110] and encodes a 130 kDa Type-1 glycoprotein composed of 976 amino acid residues [109,111]. EphA2 is localized on the cell membrane [111] and binds to ephrin-A1, -A2, -A3, -A4, and -A5 [14,22,60,112,113], but does not require ligand binding for some of its activities [114].

Like other receptor tyrosine kinases, the extracellular domain of EphA2 mediates ligand binding, which results in the autophosphorylation of its tyrosine residues [100,115]. The intracellular domain possesses intrinsic enzymatic activity, with the juxtamembrane region and the kinase domain containing many tyrosine residues that serve as docking sites for interactions with other signaling proteins containing SH2/SH3 domains [116]. The SAM domain is involved with receptor homo- or hetero-dimerization [66,69,70,71], and the PDZ-binding motif binds to other PDZ domain-containing proteins [67,68]. In our multiple sequence alignment analyses, EphA2 shows 55–70% sequence homologies and 40–50% sequence identities with other Eph receptors at the amino acid level, and the tyrosine residues are conserved within the juxtamembrane and kinase domains (data not shown).

EphA2 plays key roles in several developmental processes. Recent studies indicate that EphA2 regulates lens transparency, kidney repair following renal injury, bone remodeling, mammary gland branch morphogenesis, and inner ear development, as well as cell transformation in a variety of tumors.

### 3.1. Lens Development

Cataracts, or the opacification of the crystalline lens, are a major cause of visual impairments, accounting for up to 48% of all eye diseases [117]. Congenital cataract, in which lens deficits occur prior to or during childhood, has an estimated prevalence of 3–5/1,000 live births [118]. These early-onset cataracts may have a genetic basis, as genetic linkage studies have defined numerous mutations associated with deficits in lens development and maintenance, including those associated with connexins [119,120], crystallines [121,122,123,124,125,126,127,128,129], and intermediate-filament-like factors [130]. More recent investigations have identified the Eph family to play pivotal roles in lens development and maintenance [35,36,37,38,39,40,41,112,131,132,133,41,112,131].

#### 3.1.1. EphA2 Expression in the Lens

The first evidence that the Eph family play a role in maintaining lens clarity came from our analyses of the role of ephrin-A5 in mouse development [112,133]. EphA2 was found to be expressed in the lens fiber cell layer, most notably within the subcortical region and throughout the epithelial cell layer [35,112,131,132]. In the mature lens, EphA2 protein is detected on the shorter edges of lens fiber cells when cut into cross-sections [131]. A recent report by Son *et al*. has identified that both EphA2 and ephrin-A5 are expressed during early ocular development and continue to be expressed into postnatal stages, and that both EphA2 and ephrin-A5 also co-localize in similar locations [112,133]. Our laboratory has also observed expression of several other Eph receptors and ephrin ligands, and their extensive expression has been shown throughout lens development as early as embryonic day 12 (E12), indicating a potential role during early lens development [133]. 

#### 3.1.2. EphA2 Mutations and Cataractogenesis

Genetic analyses have shown that the 1p36 locus encodes mutated *EPHA2* in human populations with cataracts [35,36,37,38,39,40,41]. The cataract mutations in *EPHA2* reside in the intracellular compartment of the receptor, specifically within the kinase and SAM domains (Figure 1) [35,36,37,38,41]. The loss of EPHA2 function may directly or indirectly affect structural integrity, cell-cell communication, and protein stability, leading to congenital and age-related cataracts. 

The EPHA2 mutations in the kinase domain have been identified in Caucasian populations [35] and a Pakistani family [37], leading to age-related cortical cataract or autosomal recessive congenital cataract, respectively. One of the cataract mutations residing in the protein kinase domain of EPHA2, the missense mutation *Arg721Gln* (R721N), significantly alters EPHA2 signaling and cellular regulation, with significantly greater growth inhibition by ephrin-A1 [35]. Another kinase domain mutation, c.2353G > A, results in the change of alanine to threonine at codon 785 (A785T), and leads to the formation of a nuclear cataract [37]. In a recent study, a missense mutation, c.2668C > T (p.R890C), was found between the kinase domain and the SAM domain [41]. However, the underlying mechanisms by which this mutation causes cataracts are still unknown.

Additionally, four other cataract mutations have been identified in the SAM domain of EPHA2 [36,38]. A missense mutation, c.2842G > T, which substitutes a glycine with tryptophan at codon 948 (GGG > TGG: p.G948W) is associated with autosomal dominant posterior polar cataracts in Caucasians [36]. Other SAM domain mutations include a missense mutation [c.2819C > T (p.T940I) in a Chinese family], a frameshift mutation [c.2915_2916delTG (p.V972GfsX39) in a British family] and a splicing mutation [c.2826-9G > A in an Australian family] (Figure 1) [38]. Interestingly, these SAM domain mutations are autosomal dominant, suggesting that they interfere with the wild-type EPHA2 receptor functions in lens development (Table 1).

**Table 1:** EPHA2 mutations in cataractogenesis.

Mutants	Domain	Mutation	Effect	Phenotype	Ref.
c.2842G > T	SAM	Missense	G948W	Autosomal dominant posterior polar cataract	[36]
c.2819C > T	SAM	Missense	T940I	Autosomal dominant posterior polar cataract	[38]
c.2826-9G > A	SAM	Splicing	novel 71 AA	Autosomal dominant total cataract	[38]
c.2915_2916delTG	SAM	Frameshift	novel 39 AA	Autosomal dominant posterior polar cataract	[38]
c.2162G > T	Kinase	Missense	R721N	Autosomal dominant cortical cataract	[35]
c.2353G > A	Kinase	Missense	A785T	Autosomal recessive nuclear cataract	[37]
c.2668C > T	Between the kinase and the SAM	Missense	R890C	Autosomal dominant posterior cataract	[41]

#### 3.1.3. Cataract Mouse Models

The first evidence that deficiencies in Eph-ephrin signaling lead to cataract formation came from the observation that the loss of ephrin-A5 in mice resulted in lens deformations [112,133]. Ephrin-A5 knockout lenses form vacuoles in the subcortical areas starting as early as postnatal day 6 (P6), eventually becoming exacerbated at later stages and leading to lens rupture by two months of age [112]. Histological analysis of early postnatal ephrin-A5 knockout lenses before the development of serious lens pathologies show distinct alterations in the shape of lens fiber cells at P6. In the wild type, cross-sectioned fiber cells are hexagonally shaped and tightly packed into regular rows radiating from the center of the lens. In contrast, ephrin-A5 knockout lens fiber cells are mostly cuboidal and have lost this organized packing arrangement. Eph receptor expression analyses showed that EphA2 co-localized with ephrin-A5, and that the absence of ephrin-A5 reduced the tyrosine phosphorylation of EphA2, suggesting that EphA2 is one of the receptors mediating ephrin-A5 function in the lens. This notion is strongly supported by the observations both EPHA2 mutations in humans and EphA2 inactivation in mice also result in cataracts [35]. Biochemical analysis further implicated a role of ephrin-A5 in regulating adherens junction functions and the loss of ephrin-A5 could cause a reduction in adherens junction-mediated cell-cell adhesion, which may contribute to loose fiber cell packing in the lens [112].

Jun *et al.* [35] had observed that EphA2 knockout mice developed using either a secretory gene trapping strategy of partial EphA2 ectodomain fused to β-gal on FVB/NJ genetic background [134,135] or a retroviral insertion into the first intron of EphA2 gene on C57BL/6 genetic background [136] developed age-related cortical cataracts. In both strains, pathological alterations were observed one month after birth with the onset of subcapsular vacuoles in the anterior cortex, followed by lens opacity and rupture between six to eight months of age. Cataract formation in the homozygous mutant mice was over 80% by 12 months of age; in contrast, no cataract formation was observed in wild-type or heterozygous lens. EphA2 became upregulated when lens epithelial cells underwent differentiation into cortical lens fiber cells, and the amount of expression progressively decreased with age. In addition, Jun *et al*. found that the signaling molecule HSP25, a marker for oxidative stress that is overexpressed in lenses undergoing cataract formation, was upregulated in the EphA2 knockout mice [35].

Structural deformations in the surface patterning of individual fiber cells in EphA2 knockout lenses have been reported by Shi *et al*. [131]. In this study, the loss of EphA2 resulted in abnormalities in lens shape and composition, resulting in inaccurate light refraction and sutural defects in the surface patterning of individual fiber cells, indicating possible problems in lens cell migration. However, these EphA2 knockout mice were developed using an in-frame translational stop codon at exon 5 under a 129/Sv and C57BL/6 mixed background and did not develop severe cataracts, though small flecklike opacities were observed within the lens nucleus. The discrepancy of lens phenotypes in between this EphA2 knockout strain and the two others observed by Jun *et al.* [35] remains unclear, though the differences in mouse genetic background may contribute to the variability in phenotypes. 

#### 3.1.4. Signaling and Molecular Mechanisms

The recent findings on EphA2 in cataractogenesis reveal several intriguing aspects of EphA2 function and activity. Of particular interest has been the finding that four of the total known seven cataract mutations in EPHA2 are located within the SAM domain, suggesting that this domain plays a critical role in the regulation of EPHA2 function and lens development. Interestingly, all of the identified SAM domain mutations resulted in autosomal dominant phenotypes [36,38]. 

The SAM domain is a conserved protein module in several key transcription factors, scaffolding proteins and regulatory proteins that are capable of forming homo- and hetero-oligomer [71]. Previous studies have shown that the SAM domain in the Eph receptors may have multiple functions [69,70,137,138,139]. Since SAM domains facilitate protein-protein interactions, it is possible that the EphA2 SAM domain mutations may interfere with receptor oligomerization or clustering into particular complexes essential for biological signaling [65]. Both the frameshift mutation c.2915_2916delTG and splicing mutation c.2826-9G > A, which affect the EPHA2 SAM domain, significantly enhance protein-protein interactions between EPHA2 and low molecular weight protein-tyrosine phosphatase (LMW-PTP) [38], which normally associates with the *C*-terminus of the EPHA2 and negatively regulates its signaling [140,141].

Further analysis of the EPHA2 SAM domain mutations has shown that these mutations induce degradation of EPHA2 through the ubiquitin-mediated proteasomal pathway and affect their solubility [47]. The EPHA2 mutants are also incapable of promoting cell migration. However, although the mutations within the SAM domain affect EPHA2 stability and solubility, how these mutants produce autosomal dominant effects is still unknown. In addition, the role of the PDZ-binding motif on the EPHA2 *C*-terminus may also play a role in regulating receptor function, as two of the mutants, c.2915_2916delTG and c.2826-9G > A, also lack the PDZ-binding domain [38]. 

The EPHA2 kinase domain also plays an important role in regulating lens development. The R721N mutation within the kinase domain is a risk allele for cataractogenesis in human populations [35]. Biochemical analysis has shown that the R721N mutation results in higher basal activation of the EPHA2 receptor compared to wild-type EPHA2, which leads to enhanced inhibition of extracellular signal-regulated kinase (ERK) 1/2. In addition, the kinase domain missense mutation c.2353G > A has been found to cause autosomal recessive congenital cataract, though the mechanism of this mutation remains to be elucidated [37].

### 3.2. Retinal Angiogenesis

The retina contains an intricate network of vasculature whose development, organization, and maturation are tightly regulated. Initial vascularization occurs in late prenatal stages in humans and early postnatal stages in mice, with vessels forming on the superficial retinal layers from the optic nerve head and extending outward to the peripheral edges of the organ [142,143]. This peripheral expansion is followed by selective remodeling of the vascular network [142,143]. Misregulation of this process has devastating consequences that leads to pathological retinal angiogenesis, in which aberrant and deficient vessels are formed, resulting in visual impairment and blindness [142,144,145,146]. Several diseases are associated with aberrant retinal angiogenesis, including proliferative diabetic retinopathy [147,148], age-related macular degeneration (AMD) [149,150], and retinopathy of prematurity (ROP) [151], amongst others, making the understanding of this process of particular importance [145].

EphA2 has been found to be upregulated during the angiogenic process of several cancers [152,153]. Inhibition of receptor activity using an EphA2-Fc fusion protein, in which the extracellular domain of EphA2 is fused to the IgG1 Fc chain and binds with ephrin-A ligands to prevent the activation of endogenous receptors, also has been shown to inhibit vascular endothelial growth factor (VEGF)-mediated angiogenesis [154,155,156,157]. In addition, the Eph family, including members of the EphA receptor and ephrin-A ligand subgroups, has been implicated in the process of neovascularization of the retina [158].

Recent work by several groups has found EphA2 to be a promising therapeutic molecule for inhibiting retinal angiogenesis. Initial work by Chen *et al*. had found that inhibiting EphA2 activity through injection of EphA2-Fc into the rat retina inhibited retinal neovascularization, while not affecting normal retinal vascular development [159]. The targeting of EphA2 has been found to inhibit neovascularization in both the retina [29] and choroid [160]. Interestingly, ephrin-A1-Fc treatment in the eye had also been found to inhibit neovascularization, although this study implies that the inhibition is brought on by EphA2 activation and not its inhibition [161].

These results point to the targeting of EphA2 and its signaling pathway as a promising approach for the treatment of retinal angiogenesis. In particular, EphA2-Fc, which specifically targets neovascular structures while seemingly not affecting normal retinal vascular development, may hold great promise for the treatment of diseases related to retinal angiogenesis. Future studies to examine the effectiveness of targeting EphA2 and its putative ligands in the eye in the context of other retinal neovascular diseases, including diabetic retinopathy and AMD, may provide additional treatments for these debilitating diseases. Further elucidation of the mechanism by which EphA2 regulates angiogenic formation is also required.

### 3.3. Kidney

In mouse kidney organogenesis, the initiation of branching morphogenesis occurs when the membrane protrusions from the tip of the ureteric bud (UB) invades into the metanephric mesenchyme [162,163]. During development, renal epithelia arise from two distinct sources: the collecting ducts and the nephrons. The collecting ducts develop by repeated branching of the ureteric bud, and the nephrons develop by the mesenchymal-to-epithelial transition (MET) of the metanephric mesenchyme [163].

EphA2 has been shown to be expressed at high levels in the developing kidney [43]. Miao *et al*. has reported that EphA2 is expressed in the ureteric buds of embryonic kidneys using an *in vitro* three-dimensional culture system [42]. In this study, EphA2 negatively regulated hepatocyte growth factor (HGF)-induced branch morphogenesis of Madin-Darby Canine Kidney (MDCK) cells [42]. Moreover, activation of EphA2 by its ligand ephrin-A1 caused a collapse of existing branch structures and prevented new branches from forming. HGF alone induced an epithelial-to-mesenchymal transition (EMT) which is required for the rearrangement and remodeling of MDCK cells during branch morphogenesis. In contrast, EphA2 reversed this process, which ensured that branch morphogenesis occurred within the correct location. Consistent with this observation, treatment with ephrin-A1 also binds to MDCK cell compaction [164].

In addition to kidney development, EphA2 has been found to play roles in Renal Ischemia-Reperfusion Injury (IRI), which is a major cause of acute kidney injuries in both native kidneys and renal allografts. Studies in several models have suggested that one of the key events in the pathogenesis of IRI is the disruption of the tubular epithelial actin cytoskeleton [165,166,167,168]. In previous studies, Eph receptors have been found to be a key developmental regulators of cytoskeletal remodeling during embryonic development, particularly in respect to the vascular and central nervous systems [12,13,14,23,24,25,26,27,28,29,169]. However, the contribution of surface receptors that orchestrate cytoskeletal repair is not fully understood in the repair process of kidney following IRI.

Baldwin *et al*. [89] was the first to show that EphA2 is a critical regulator of actin dynamics in a mouse model of renal IRI. While basal expression of EphA2 protein was observed in distal tubular segments, IRI resulted in more intense and generalized up-regulation of EphA2 protein expression. This increase in EphA2 expression appears to be through a Src kinase-dependent pathway, as the enhanced expression of EphA2 was inhibited by the Src kinase inhibitor PP2. Src kinases also strongly activated the human EphA2 promoter, suggesting that this up-regulation occurs through a transcriptional mechanism. In addition, ephrin-A1 treatment led to tyrosine phosphorylation of EphA2, and this particular interaction between up-regulated EphA2 and its putative ligand may serve to enhance cell contact-dependent signaling for cytoskeletal repair in renal IRI.

Recent studies have also reported the increase of EphA2 expression under stressful conditions. Specifically, EphA2 up-regulation has been investigated in cultured Inner Medullar Collecting Duct (IMCD-3) cells and in the renal medulla in response to urea stress and hypertonicity [43,170]. EphA2 was expressed in high levels within the collecting ducts of the renal papilla and the renal medulla but only in low levels within rat renal cortex [43]. An enhanced expression of EphA2 has also been observed in the livers of rats stimulated with bacterial lipopolysaccharides [171] and in epithelial cells exposed to the detergent deoxycholic acid [172]. Together, these studies suggest that EphA2 has a critical role in the tissue repair process of disease models, such as IRI. More specifically, EphA2 may behave as an injury- and stress-responsive regulator.

### 3.4. Bone

Ephs and ephrins are expressed in chondrocytes, osteoclasts and osteoblasts, as well as endothelial cells and neurons in the bones and in the bone marrow [19,20]. Bones are constantly remodeled throughout life, and the coupling of bone resorption and formation is tightly regulated by communication between osteoclasts and osteoblasts [173]. Osteoclasts are multinucleated cells responsible for bone resorption through cell-cell fusion [174]. Unlike osteoclasts, bone-forming osteoblasts are mononuclear cells derived from mesenchymal progenitors and express the two major membrane-bound proteins, macrophage-colony stimulating factor (M-CSF) and the receptor activator of NF-κB ligand (RANKL) [175,176]. During differentiation of cultured osteoclasts induced by RANKL, the EphA1, A2 and A4 receptors, and the ephrin-A2, -B1, and -B2 ligands, are clearly expressed [20,21,22].

Irie *et al*. have recently found that bidirectional EphA2-ephrin-A2 signaling is critical in bone remodeling at the initiation phase [22]. The EphA2-ephrin-A2 interaction promoted bone resorption and concomitantly suppressed osteoblastogenesis in a manner distinct from EphB4-ephrin-B2 interaction [21]. In addition, the loss of EphA2 enhanced osteoblastogenesis by a decrease in GTP-RhoA, suggesting that signaling through EphA2 into osteoblasts suppresses osteoblast differentiation by activating RhoA.

### 3.5. Mammary Gland Branch Morphogenesis

Mammary gland branching morphogenesis is a developmental process during which an extensive complex of the growing ducts forms from a rudimentary epithelial bud [177,178]. Expressions of some Eph family receptors and their ligands have been reported in the mammary gland [34,45,46,51,179,180,181,182,183]. In initial developmental studies, two receptor protein tyrosine kinases, EphB4 and EphA2, were originally isolated at the RNA level from the mature mouse mammary glands [34]. The EphB4 receptor is expressed in myoepithelial luminal cells and developmentally regulated in a hormone-dependent manner during normal mammary gland development, with misregulation resulting in the formation of invasive mouse mammary tumors. Further studies have found that ephrin-B2 is expressed on the luminal cells in the mammary gland and acts as a cognate ligand of EphB4 [183]. Estrogen acts as one of the mediators to induce the expression of EphB4 and ephrin-B2 [183].

More recently, a genome-wide transcript analysis has identified only the Eph family molecules EphA2 and ephrin-B1 in the terminal end buds forming at the tips of the ducts [44]. EphA2 expression was also shown to be developmentally controlled in the growth and branch morphogenesis of normal mammary epithelium [34,44]. Loss of EphA2 resulted in reduction of mammary epithelial proliferation and marked inhibition of epithelial branching necessary for complete fat pad filling [46]. Further *in vitro* analyses have shown that EphA2 expression was negatively regulated by estrogen and c-myc [184]. EphA2 expression was also detected in human mammary epithelial cells [45,153,185]. EphA2 leads to growth arrest and differentiation in normal human mammary epithelial cells in three-dimensional cultures, and its level was significantly decreased in the differentiated cells [186], whereas the expression increased in breast cancer [187]. Interestingly, enhanced EphA2 expression in human breast cancer is associated with a poor patient prognosis [188].

Epithelial branch morphogenesis is a fundamental biological process by which the endocrine hormones and local paracrine interaction between the developing epithelial ducts and their adjacent mesenchymal stroma drives mammary gland development [46,51]. Some of the cytockines and growth factors, such as FGF, HGF and TGF-β, act as critical molecules in the local regulation of branch morphogenesis [182,189]. Vaught *et al*. [46] had found that HGF-induced mammary epithelial branch morphogenesis was significantly reduced in EphA2-deficient cells relative to wild-type controls, which correlated with increased RhoA activity. Taken together, these results indicate that EphA2 functions as a positive regulator in mammary gland morphogenesis.

### 3.6. Ear

The mammalian inner ear initially forms an epithelial sac-like otocyst, and differentiates into a highly complex structure. They are subdivided into the auditory component (the coiled, snail-shaped cochlea, that regulates hearing), and five vestibular regions (consisting of the saccular and utricular maculae, and the ampullar cristae of the three semicircular canals whose primary role is to regulate balance) [190]. Several studies have reported the expression of the Eph family in structures of the inner ear [48,190,191,192]. Ephrin-A1 and ephrin-A2 were detected in the epithelial cells lining the fluid filled ducts and in connective tissue regions of the inner ear, respectively [190]. EphA4 was expressed in vestibular hair cells, and EphA5 and EphA7 were exhibited in cochlear and vestibular supporting cells, suggesting that these Eph receptors have a role in establishing the formation and cellular organization of the inner ear [190]. A later study by Pickles *et al*. had also identified the expression of EphA4 and its ligand ephrin-A2 in cochlear tissues [191]. In addition, disruption of EphB1 or EphB3 receptors in mice lead to deficits in cochlear function [193]. These results indicate that specific Eph receptors are necessary for cochlear function in the inner ear. 

Recent examinations of the function of the Eph family during development have identified the expression of EphA2 during otic placode formation between E8.5 and E10.5 [48]. The vertebrate inner ear develops from the otic placode, an ectodermal thickening located adjacent to the posterior hindbrain [194]. Saeger *et al*. first observed EphA2 expression in the otic region in the ventral and posterior ectoderm; the ventral, posterior and subplacodal mesenchyme; and the anterior neural tube (rhombomere r4) [48]. However, EphA2 involvement in inner ear development remains to be functionally demonstrated.

## 4. Conclusions and Perspectives

The Eph receptors and ephrin ligands are expressed during the development of a range of vertebrate species. Critical roles of EphA2 have been shown in lens development and renal repair. However, key questions remain in terms of how EphA2 and its ephrin-A ligands cooperate for tissue patterning. The role of EphA2 in the lens has been of particular interest in the past several years, resulting in several advances in understanding its role in lens biology. Among the members of the Eph family, only EphA2 and the ligand ephrin-A5 have thus far been identified as critical regulators in lens development. Both the EphA2 kinase and SAM domains are required for function. In particular, the analysis of human EPHA2 SAM domain mutations reveals a novel mode of action of SAM domain, namely the regulation of protein stability and solubility. Additionally, molecular and signaling mechanisms underlying EphA2 function within the lens are beginning to come to light. However, many important questions remain. Thus far, most of the signaling pathway studies have utilized *in vitro* models, but how this applies to lens tissues remain to be determined. Functional studies using transgenic knock-in mouse models expressing various EPHA2 signaling mutations may provide further insights. The importance of EphA2 in other tissues, including the retina, kidney, bone and ear development, has also been recently reported, and many of these studies are still in their infancy. The questions concerning the intracellular pathways linking EphA2 receptor and ephrin-A signaling still need to be analyzed, especially in relation to other signals in organ development.
